# Adherence to the American Cancer Society Guidelines for Cancer Survivors and Health-Related Quality of Life among Breast Cancer Survivors

**DOI:** 10.3390/nu11122924

**Published:** 2019-12-03

**Authors:** Dahye Koh, Sihan Song, Sang-Eun Moon, So-Youn Jung, Eun Sook Lee, Zisun Kim, Hyun Jo Youn, Jihyoung Cho, Young Bum Yoo, Se Kyung Lee, Jeong Eon Lee, Seok Jin Nam, Jung Eun Lee

**Affiliations:** 1 Department of Food and Nutrition, Seoul National University, Seoul 08826, Korea; yaong0516@snu.ac.kr (D.K.); songsihan@snu.ac.kr (S.S.); fnmse@snu.ac.kr (S.-E.M.); 2 Research Institute and Hospital, National Cancer Center, Goyang 10408, Korea; goje1@ncc.re.kr (S.-Y.J.); eslee@ncc.re.kr (E.S.L.); 3 Department of Surgery, Soonchunhyang University Bucheon Hospital, Soonchunhyang University College of Medicine, Bucheon 14584, Korea; sk4091@hanmail.net; 4 Department of Surgery, Chonbuk National University Medical School, Jeonju 54907, Korea; yhj0903@jbnu.ac.kr; 5 Department of Surgery, Keimyung University School of Medicine, Daegu 42601, Korea; chojh0404@hanmail.net; 6 Department of Surgery, Konkuk University Medical Center, Seoul 05030, Korea; 0117652771@kuh.ac.kr; 7 Department of Surgery, Samsung Medical Center, Sungkyunkwan University School of Medicine, Seoul 06351, Korea; sekyung.lee@samsung.com (S.K.L.); jeongeon.lee@samsung.com (J.E.L.); seokjin.nam@samsung.com (S.J.N.); 8 Research Institute of Human Ecology, Seoul National University, Seoul 08826, Korea

**Keywords:** breast cancer survivors, health-related quality of life (HRQoL), cancer survivor guidelines, physical activity

## Abstract

The development and validation of guidelines for breast cancer survivors are of importance due to the increased survival rate for breast cancer. In this cross-sectional study, we aimed to examine the association between adherence to the American Cancer Society (ACS) guidelines for cancer survivors and health-related quality of life (HRQoL). A total of 618 breast cancer survivors aged 30 to 81 years who had been diagnosed with stage I to III primary breast cancer and had surgery at least a year before enrollment were included. The participants completed the 36 Item Short-Form Health Survey (SF-36) to evaluate HRQoL, and adherence scores were calculated based on the Nutrition and Physical Activity Guidelines for Cancer Survivors, which were released by the ACS. Increasing adherence scores were associated with increasing scores on the physical component summary (PCS) and the physical functioning (PF), bodily pain (BP), and vitality (VT) domains (*p* for trend <0.001 for PCS and PF, 0.01 for BP, and 0.02 for VT); these scores were mainly driven by the associations among survivors with stage II–III cancer. Further prospective studies are needed to evaluate whether adherence to these guidelines improves HRQoL scores among breast cancer survivors.

## 1. Introduction

Breast cancer is the most common cancer (24.2% of the total cases in 2018) and the leading cause of cancer deaths (15.0% of the total cancer deaths in 2018) in women worldwide [1]. Similarly, it is the most common female cancer in Korea, with an age-standardized incidence rate among women of 54.9 per 100,000 in 2016 [2]. Due to advances in the early detection and treatment of breast cancer, the survival rate of patients has continuously increased in Korea. According to Korea Central Cancer Registry (KCCR) data, the 5 year relative survival for Korean women with breast cancer between 2012 and 2016 was 92.7%, which was 14.8% higher than that of those diagnosed between 1993 and 1995 [2]. Various lifestyle factors, especially nutrition and physical activity, may be associated with a better prognosis and health-related quality of life (HRQoL) for breast cancer survivors.

Among breast cancer survivors in the U.S. Health, Eating, Activity, and Lifestyle (HEAL) study, better post-diagnostic diet quality was directly associated with improved physical and mental functioning [3]. In addition, several observational studies examined the possible relationship between physical activity and quality of life in Greek [4] and Korean [5] breast cancer survivors and showed that engaging in physical activity was positively associated with a better quality of life, such as fewer depressive symptoms and less fatigue and pain. Furthermore, some intervention studies also support the positive effects of a healthy diet and exercise [6,7,8] or increased physical activity [9,10] on the quality of life in breast cancer survivors in mainly Caucasian populations. Asian breast cancer patients have different characteristics, including age of onset, tumor types, and menopausal status at diagnosis, compared to Western breast cancer patients [11,12]. Nonetheless, most of these studies were conducted in Western populations [3,4,6,7,8,9,10], and there is a lack of evidence about associations between healthy diet and quality of life among cancer survivors in Asian populations. 

In addition to increasing attention on the role of diet and physical activity in the health status of cancer survivors, several guidelines for cancer survivors to achieve a better prognosis and quality of life have been suggested. In 2007, the World Cancer Research Fund/American Institute for Cancer Research (WCRF/AICR) reported that cancer survivors should follow recommendations for cancer prevention regarding foods, body fatness, and physical activity [13]. However, the evidence regarding the effects of nutrition and exercise in cancer survivors reviewed in the report was inconclusive. The WCRF/AICR has continuously updated scientific research and recommendations on breast cancer prevention and survivorship regarding nutrition and physical activity, and presented the results in 2018 [14,15]. Although the WCRF/AICR suggested some evidence of links between lifestyle factors (i.e., physical activity, foods containing fiber, and soy products) and better survival after a breast cancer diagnosis, this evidence was included in the limited evidence category [15]. The American Cancer Society (ACS) released guidelines on nutrition and physical activity for cancer prevention and highlighted the importance of weight management, physical activity, and diet [16]. In 2012, the ACS advised that cancer survivors should maintain a healthy body weight, engage in physical activity, and achieve a healthy diet based on the ACS guidelines for cancer prevention [17]. Furthermore, the ACS and the American Society of Clinical Oncology additionally released breast cancer survivorship care guidelines including nutrition, physical activity, and clinical care recommendations [18]. 

Although compliance with the guidelines for a healthy lifestyle may be beneficial to breast cancer survivors, the evidence has not yet been clearly established. Several epidemiological studies have suggested that better adherence to guidelines for cancer prevention was associated with a reduction in breast cancer incidence and mortality [19,20,21,22]. Nonetheless, few studies have examined the possible relationship between adherence to guidelines for breast cancer survivors and quality of life [23,24,25,26]. 

In this cross-sectional study, we aimed to determine whether increasing adherence to the ACS guidelines for cancer survivors was associated with increasing levels of HRQoL scores among Korean breast cancer survivors. In addition, we examined the associations between each component of the ACS guidelines and HRQoL, and whether these associations varied by breast cancer stage at diagnosis.

## 2. Materials and Methods 

### 2.1. Study Population 

Female breast cancer survivors who underwent breast cancer surgery at least a year before enrollment at six hospitals in Korea between June 2015 and May 2019 were recruited, including 656 participants who had been diagnosed with stage I to III breast cancer according to the American Joint Committee on Cancer (AJCC), and did not have metastasis or recurrence of breast cancer before enrollment. Those participants were asked to complete the 36 Item Short-Form Health Survey (SF-36) to evaluate HRQoL. Breast cancer survivors were excluded if they had any other cancers before enrollment or did not report that information (*n* = 17), who did not have body mass index (BMI) (*n* = 1) or dietary information (*n* = 8), or who did not complete the SF-36 questionnaire (*n* = 6). Study participants who reported implausible energy intake (±3 standard deviations (SDs) from the mean of the log-transformed energy intake, *n* = 6) were additionally excluded. In total, 618 breast cancer survivors were included in the analysis and all participants provided written informed consent. The institutional review boards (IRBs) of each hospital approved all procedures of this study: the National Cancer Center, Korea (NCC2014-0101), Soonchunhyang University Hospital (SCHBC2014-12-004-001), Chonbuk National University Hospital (CUH2014-05-002-005 and CUH2018-02-004-004), Keimyung University Dongsan Medical Center (DSMC2015-03-026), Konkuk University Medical Center (KUH1020068), and Samsung Medical Center (SMC2016-07-073-004).

### 2.2. Data Collection

Dietary information was collected using either 3-day dietary records or food frequency questionnaires (FFQs). A total of 338 breast cancer survivors reported their dietary intake using 3-day dietary records on three nonconsecutive days, including two weekdays and one day on the weekend. The participants recorded all foods and beverages using food photograph booklets provided to help them estimate portion size. Food and nutrient intake from 3-day dietary records were calculated using the Computer-Aided Nutritional Analysis Program (CAN-pro) version 4.0 (The Korean Nutrition Society, Seoul, Korea). The daily intake (g/day) of each food item was calculated by averaging the 3-day intakes. An FFQ was developed for Korean breast cancer survivors in 2016, which is composed of 123 food and beverage items [27] and was completed by 280 participants. Energy and nutrient intakes were calculated by multiplying the portion size by the daily frequency; the nine possible frequency categories were never or almost never, once per month, two to three times per month, once per week, two to four times per week, five to six times per week, once per day, two times per day and three times per day. In the FFQ validation study, the median energy-adjusted Pearson correlation coefficients were 0.41 for macronutrients and 0.36 for micronutrients (manuscript in preparation).

Anthropometric data (height and weight) were measured at enrollment to calculate BMI (kg/m^2^). The height and weight measured at diagnosis were used if the information at enrollment was missing. For physical activity levels, the data were collected using a structured questionnaire containing the type, duration, and frequency of exercise in which the participants regularly engaged. A metabolic equivalent (MET)-hours/week was calculated for each type of exercise and summed over all activities to yield the total MET-hours/week. A MET value was assigned to each physical activity reported in the questionnaire according to the Compendium of Physical Activities [28]. Other factors, such as smoking status, alcohol intake, socioeconomic status, and reproductive history, were also collected through the questionnaire and clinical information was collected via the medical records from each hospital. 

HRQoL levels were assessed using the SF-36 health survey version 2.0, a multipurpose health survey with 36 questions that yield an eight-scale profile of scores for physical and mental health measures [29]. The eight domains are physical functioning, role-physical, bodily pain, general health, vitality, social functioning, role-emotional, and mental health, and the results are presented in the form of summaries of the two main domains: the physical component summary, and the mental component summary [30]. The SF-36 profile scales were calculated based on the calculation manual provided by the developers of the survey using the Pro CoRE version 1.3 Smart Measurement System (Optum Inc., Johnston, RI, USA). The physical component summary and mental component summary were calculated from all health domains; physical functioning, role-physical, bodily pain, and general health contributed to the physical component summary, while vitality, social functioning, role-emotional and mental health contributed to the mental component summary [30,31]. The higher the scores, the better the status for all the domains of HRQoL [29].

### 2.3. Adherence Scores

Adherence scores were calculated based on each criterion of the Nutrition and Physical Activity Guidelines for Cancer Survivors, which was released by the ACS [17] (Table 1). The ACS guidelines for cancer survivors contain the following three elements: (1) achieving and maintaining a healthy body weight, (2) engaging in regular physical activity, and (3) following the ACS Guidelines on Nutrition and Physical Activity for Cancer Prevention (i.e., achieving a dietary pattern that is high in vegetables, fruits, and whole grains and low in processed and red meat) [16,17].

First, BMI categories were based on the Asia-Pacific classification for obesity: underweight (<18.5 kg/m^2^), normal (18.5 to <23 kg/m^2^), overweight (23 to <25 kg/m^2^), moderate obese (25 to <30 kg/m^2^), and severe obese (≥30 kg/m^2^) [32]. As both underweight [33] and obesity [34] have been suggested to worsen the prognosis of breast cancer patients, we assigned the lowest score to <18.5 or ≥30 kg/m^2^, and participants with a BMI of 25 to <30, 23 to <25, and 18.5 to <23 kg/m^2^ received a score of 2, 3, and 4, respectively. Second, the participants were divided into four groups based on quartiles of physical activity levels, and the highest quartile was given a score of 4, whereas the lowest quartile was given a score of 1. Third, all food items obtained from dietary records and FFQs were grouped into fruits and vegetables, whole grains, and red and processed meat. For each food group, the intake (g/day) was divided into quartiles, and a score of 4 was assigned to the highest quartile of fruits and vegetable intake and whole grains intake and the lowest quartile of red and processed meat intake. Each score from the three food groups was then summed and regrouped into quartiles with the lowest and the highest quartile given a score of 1 and 4, respectively. The overall adherence scores were calculated by summing the three scores of adherence to the ACS guidelines and ranged from 3 to 12.

### 2.4. Statistical Analysis 

The least-squares means (LS-means) and 95% confidence intervals (CIs) of HRQoL were calculated according to the quintiles of adherence scores or physical activity levels using the generalized linear models (GLMs). The models were adjusted for age (years, continuous), energy intake (kcal/day, continuous), menopausal status at diagnosis (premenopausal or postmenopausal), stage (I, II, or III), time since surgery (1 to <2 years, 2 to <5 years, or ≥5 years), education level (elementary school or below, middle school, high school, or college or above), dietary supplement use (yes or no), alcohol intake (current, past, or never), smoking status (never or ever), and center. We also conducted subgroup analyses to examine the associations by breast cancer stage at diagnosis (I and II–III) and by menopausal status at diagnosis (premenopausal and postmenopausal). Missing data (<1%) on education levels, dietary supplement use, and alcohol intake were assigned to the most frequent category. Missing data on smoking status (4%) were assigned to the never smoking category. The statistical significance of interaction terms was estimated using the Wald test of the cross-product terms of the adherence scores (or physical activity levels) and interaction variables. All statistical tests were two-sided, and *p*-values less than 0.05 were regarded as statistically significant. Multiple comparisons were additionally adjusted using a false discovery rate (FDR) and considered statistically significant for *p*-values < 0.1. SAS version 9.4 (SAS Institute, Cary, NC, USA) was used for all analyses. 

## 3. Results

Table 2 shows the characteristics of study participants by adherence scores. The mean (SD) values for the age, BMI, and physical activity of the participants was 52.37 (8.29) years, 23.33 (2.97) kg/m^2^, and 33.02 (33.96) MET-hours/week, respectively. Over 60% of breast cancer survivors were premenopausal at diagnosis (65.86%), married or cohabiting (79.77%), and dietary supplement users (63.89%). Most participants never smoked and did not drink alcohol at enrollment. Approximately half of the breast cancer survivors had been diagnosed with stage I cancer, and over 70% were enrolled less than 5 years after breast cancer surgery.

We observed that increasing adherence scores were associated with increasing levels of physical component summary, physical functioning, bodily pain, and vitality scores in all breast cancer survivors in this study (Table 3); LS-means (95% CIs) of the lowest and the highest quintiles of adherence scores were 46.29 (44.76–47.83) and 48.99 (47.34–50.63; *p* for trend < 0.001), respectively, for physical component summary, 44.08 (42.47–45.69) and 47.30 (45.58–49.03; *p* for trend < 0.001), respectively, for the physical functioning, 47.27 (45.25–49.29) and 49.91 (47.74–52.08; *p* for trend = 0.01), respectively, for bodily pain, and 45.37 (43.05–47.68) and 48.56 (46.07–51.04; *p* for trend = 0.02), respectively, for vitality. These associations remained statistically significant after adjusting for multiple comparisons with the FDR method.

When stratified by breast cancer stage at diagnosis, the significant associations between adherence scores and HRQoL were limited to breast cancer survivors with stage II–III cancer (Table 4). Among participants with stage II–III cancer, high adherence to the ACS guidelines was associated with higher scores for the physical component summary (*p* for trend < 0.001), physical functioning (*p* for trend < 0.001), role-physical (*p* for trend = 0.03), bodily pain (*p* for trend < 0.001), and vitality (*p* for trend = 0.01), and these associations remained statistically significant after adjusting for multiple comparisons. The results revealed a significant interaction by breast cancer stage (stage I and II–III) for the physical component summary (*p* for interaction = 0.001), role-physical (*p* for interaction = 0.005), bodily pain (*p* for interaction = 0.001), and social functioning (*p* for interaction = 0.03) domains. 

We further examined whether physical activity alone was associated with HRQoL scores, and the association varied by cancer stage at diagnosis. Breast cancer survivors with higher physical activity levels were more likely to have higher HRQoL scores (Table 5); LS-means (95% CIs) of the lowest and the highest quintiles of physical activity levels were 46.93 (45.35–48.51) and 49.35 (47.64–51.06; *p* for trend = 0.001), respectively, for the physical component summary, 44.93 (43.27–46.60) and 47.47 (45.67–49.26; *p* for trend = 0.003), respectively, for physical functioning, 48.09 (46.02–50.17) and 50.37 (48.12–52.61); *p* for trend = 0.02), respectively, for bodily pain, 45.62 (43.63–47.62) and 48.03 (45.87–50.18; *p* for trend = 0.001), respectively, for general health, and 46.52 (44.16–48.88) and 50.03 (47.47–52.58; *p* for trend < 0.001), respectively, for vitality. These associations were all significant after applying an FDR <0.1. Increased levels of vitality (*p* for trend = 0.003) among breast cancer survivors with stage I cancer and the physical component summary (*p* for trend = 0.01), physical functioning (*p* for trend = 0.04), bodily pain (*p* for trend = 0.03), and general health (*p* for trend = 0.01) among those with stage II–III cancer were associated with increased physical activity levels after adjustment for multiple comparisons (Table 6).

Subgroup analyses were conducted to examine the associations between adherence scores with HRQoL by menopausal status at diagnosis (premenopausal and postmenopausal), as shown in Table S1. The significant associations were limited to premenopausal women; higher adherence scores were associated with higher scores for the physical component summary (*p* for trend = 0.001), physical functioning (*p* for trend < 0.001), bodily pain (*p* for trend = 0.04), and vitality (*p* for trend = 0.01), and these associations were all significant after applying an FDR < 0.1 (Table S1). When we examined whether the association between physical activity levels and HRQoL was modified by menopausal status at diagnosis, we found that increasing physical activity levels were associated with increasing scores for the physical component summary (*p* for trend = 0.01) and the physical functioning (*p* for trend = 0.01), general health (*p* for trend = 0.03) and vitality (*p* for trend = 0.01) domains among premenopausal survivors and general health (*p* for trend = 0.005) among postmenopausal survivors after adjustment for multiple comparisons using the FDR method (Table S2).

We examined the associations of BMI and diet scores separately with HRQoL by breast cancer stage. Higher BMI scores were associated with an increasing score for the physical component summary (*p* for trend = 0.01) and the physical functioning (*p* for trend = 0.04), role-physical (*p* for trend = 0.03), and bodily pain (*p* for trend = 0.02) domains among breast cancer survivors with stage II–III cancer (Table S3). These associations were all significant after applying an FDR < 0.1. The association was not statistically significant among those with stage I cancer. The results revealed a significant interaction by breast cancer stage (stage I and II–III) for the physical component summary (*p* for interaction = 0.05) and vitality (*p* for interaction = 0.05) domains. Higher diet scores were associated with increased physical component summary (*p* for trend = 0.01), physical functioning (*p* for trend = 0.01), role-physical (*p* for trend = 0.01), bodily pain (*p* for trend = 0.01), vitality (*p* for trend = 0.01), social functioning (*p* for trend = 0.05), and role-emotional (*p* for trend = 0.06) among breast cancer survivors with stage II–III cancer, with statistical significance at FDR < 0.1 (Table S4). We found significant interaction by breast cancer stage (stage I and II–III) for the physical component summary (*p* for interaction = 0.01), role-physical (*p* for interaction = 0.001), bodily pain (*p* for interaction = 0.001), social functioning (*p* for interaction = 0.03), and role-emotional (*p* for interaction = 0.03).

## 4. Discussion

The purpose of our study was to examine whether adherence to the ACS guidelines was associated with HRQoL among Korean breast cancer survivors. We found that increasing adherence scores were associated with higher scores for the physical component summary and the physical functioning, bodily pain, and vitality domains among the SF-36 scales. When the participants were stratified by breast cancer stage at diagnosis (I and II–III), positive associations between adherence to the ACS guidelines and HRQoL were observed only in the participants with stage II–III cancer. As cumulative evidence has suggested the benefit of physical activity on the quality of life among cancer survivors, we examined whether physical activity alone was associated with HRQoL and found that increasing physical activity was associated with increasing levels of physical component summary, physical functioning, bodily pain, general health, and vitality scores among the HRQoL components. In addition, significant associations of BMI and diet scores with HRQoL were limited to breast cancer survivors with cancer stage II–III.

The findings of the present study are consistent with our previous research and other observational studies [3,4,5,23,24,26,35,36]. We found that Korean breast cancer survivors with greater adherence to the ACS guidelines had higher levels of social functioning, which was assessed by a validated Korean version of the European Organization for Research and Treatment of Cancer Quality of Life Questionnaire Core 30 (EORTC QLQ-C30) [24]. In our previous cross-sectional study, higher physical activity levels were associated with a better quality of life in terms of fatigue, pain, and sexual functioning among Korean women who had been diagnosed with breast cancer [5]. In another cross-sectional study that we conducted, two dietary patterns, the healthy dietary pattern and the Western dietary pattern, were empirically derived using factor analysis among Korean breast cancer survivors, and higher healthy dietary pattern scores were associated with decreasing dyspnea and increasing insomnia scores [35]. A prospective cohort study in Hong Kong involving 1,462 Chinese breast cancer survivors investigated the association between adherence to the WCRF/AICR guidelines and HRQoL and showed that greater adherence to cancer prevention recommendations was related to higher scores for global health status, physical functioning, and role functioning, and lower scores for fatigue and pain [23]. Among elderly female breast cancer survivors in the Iowa Women’s Health Study, the participants who adhered to the WCRF/AICR diet and physical activity guidelines had better physical and mental component summary scores compared to those who did not follow the guidelines [26]. In a cross-sectional study which was nested in the HEAL cohort study, breast cancer survivors with better diet quality, assessed using the Diet Quality Index, had higher scores for physical functioning, bodily pain, social functioning, role-emotional, mental health, and the mental component summary among the SF-36 scales [3]. However, there was no significant association between the Healthy Eating Index-2010 (HEI-2010) scores and quality of life in a cross-sectional study of 44 postmenopausal breast cancer survivors [36]. Greek breast cancer survivors who had higher physical activity levels also had increased self-esteem and a better quality of life, including physical, role, emotional, cognitive, and social functioning, as well as decreased anxiety and depressive symptoms [4].

Furthermore, several intervention studies support that healthy lifestyle behaviors (maintaining a healthy body weight, engaging in exercise, or consuming a healthy diet) improved cancer survivors’ quality of life [6,8,37,38,39]. The Programme of Accompanying women after breast Cancer treatment completion in Thermal resorts (PACThe) trial also showed that SF-36 physical and mental subscores improved among breast cancer survivors in France who participated in group physical training and nutritional education, yet this improvement was not significant after a year [6]. The ENERGY trial, a randomized trial with an intensive program group versus an attention control group that was advised to adhere to the ACS dietary and exercise guidelines, found poorer physical function and symptoms in the control group compared to the intervention group and greater improvement in vitality in the intervention group compared to the control group at 6 months [8]. A randomized controlled trial of 12-month home-based diet and exercise found that physical activity and dietary intervention moderated the rate of decline in SF-36 social functioning and increased mental health among survivors of colorectal, breast, and prostate cancer [37]. In the U.S. intervention study, lifestyle modification interventions regarding nutrition, physical activity, and stress management in cancer survivors (mostly breast, prostate, or skin cancer) improved quality of life [38]. Likewise, the Adapted Physical Activity and Diet (APAD) intervention in patients with early breast cancer improved fatigue and quality of life at the 1 year follow-up [39]. 

Although it remains unclear how healthy lifestyle behaviors contribute to the improvement in quality of life in cancer survivors, modifications in insulin-like growth factor (IGF) actions [40,41,42,43] and decreased insulin resistance [44,45] may be potential mechanisms. The potential link of lifestyle factors to breast cancer risk or prognosis through IGF-1 signaling and the insulin resistance pathway has been suggested in several studies [40,41,42,43,44,45]. A few intervention studies on breast cancer survivors showed that a healthy diet and exercise modified the levels of IGF-1 and insulin resistance-related markers [40,44]. A randomized controlled trial of postmenopausal breast cancer survivors found that aerobic exercise decreased IGF-1 and insulin-like growth factor-binding protein-3 (IGFBP-3), a predominant binding protein for IGF-1 [40]. Moreover, a 12 week diet and exercise intervention in overweight and obese breast cancer survivors resulted in significant improvements in the homeostasis model assessment of insulin resistance (HOMA-IR) [44]. Furthermore, several epidemiologic studies discovered an increased risk of breast cancer with higher levels of IGF-1 and the ratio of IGF-1 to IGFBP-3 [41,42]. Indeed, a pooled meta-analysis of nested case-control studies found a relative risk of 1.2 for breast cancer risk comparing top to bottom categories of IGF-1 levels [43]. Elevated HOMA scores were also associated with higher breast cancer mortality among breast cancer survivors [45].

Several epidemiologic studies found that a healthy lifestyle was associated with improved survival regardless of cancer stage or menopausal status [20,46,47]. We did not find any significant association of adherence scores with HRQoL among breast cancer survivors who were postmenopausal at diagnosis, but more components of HRQoL reached statistical significance among those with stage II–III cancer or who were premenopausal at diagnosis. In addition, physical activity was associated with HRQoL similarly across cancer stages and menopausal status at diagnosis. Although the reasons are unclear, it is possible that behavioral changes are stronger determinants of quality of life among young Korean breast cancer survivors with stage II–III cancer than among those with stage I cancer. Motivation and self-concept may vary by cancer stage, but there are limited studies on Korean breast cancer survivors. Further prospective studies in Asian populations are warranted to examine whether the associations for lifestyle factors differ by stage and menopausal status at diagnosis.

In the present study, increased adherence to the ACS guidelines was associated with the physical component summary, the two components of physical HRQoL, and vitality, a component of the mental HRQoL, but not with the mental component summary of the HRQoL, which may have been due to measurement errors in the mental components because the HRQoL levels of the SF-36 questionnaire were self-reported. Hence, further investigation in Asian breast cancer survivors is needed. 

This study suggests the significance of adherence to healthy lifestyle behaviors for a better quality of life for breast cancer survivors. However, the results of our study should be interpreted cautiously due to several study limitations. First, because this was a cross-sectional study, a causal relationship between adherence to guidelines for cancer survivors and HRQoL could not be determined. Further prospective studies are warranted to evaluate a temporal relationship between adherence to guidelines and HRQoL scores. Second, we obtained dietary information using either 3-day dietary records or the FFQs. However, we did not find an appreciable difference by the dietary measurement method. Third, measurement errors inherent in the dietary assessment may be present. However, because 3-day dietary records and a validated FFQ were used, measurement errors may not fully explain our findings. Fourth, residual confounding might exist in our study, but we adjusted for possible confounding factors, including smoking, alcohol intake, and cancer stage. Lastly, although the generalizability of our results to breast cancer survivors in Korea may be limited, we believe that generalizability may not be such a problem in our study, as the participating hospitals treated many patients from all over the country. 

## 5. Conclusions

In conclusion, increasing adherence to the ACS guidelines for cancer survivors was associated with better HRQoL among Korean breast cancer survivors, and this positive association between adherence scores and HRQoL scores was more pronounced among breast cancer survivors with stage II–III cancer compared to those with stage I cancer. In addition, increasing physical activity was independently associated with higher HRQoL scores. Our study suggests the importance of adherence to healthy lifestyle behaviors for a better quality of life among Korean breast cancer survivors and emphasizes the necessity of future prospective studies regarding modification of lifestyle factors and quality of life or cancer outcomes.

## Figures and Tables

**Table 1:** The ACS guidelines criteria and operationalization of adherence scores.

The ACS Guidelines for Cancer Survivors	Operationalization	Scoring
1. Achieving and maintaining a healthy body weight	BMI (kg/m^2^)	
	<18.5 or ≥30, 25–<30, 23–<25, and 18.5–<23	1–4
2. Engaging in regular physical activity	(MET-hours/week)	
	Quartile 1–4	1–4
3. Following the ACS guidelines for cancer prevention: achieving a dietary pattern that is high in vegetables, fruits, and whole grains and low in processed and red meat *.	Fruits and vegetables intake (g/day)	
	Quartile 1–4	1–4
	Whole grains intake (g/day)	
	Quartile 1–4	1–4
	Red and processed meat intake (g/day)	
	Quartile 1–4	4–1

Abbreviations: ACS, American Cancer Society; BMI, body mass index; MET, metabolic equivalent task. * We summed the scores of three groups and divided these scores into quartiles (1–4).

**Table 2:** Characteristics of study participants according to adherence scores.

		Quintiles of the Adherence Scores				
	All	Q1	Q2	Q3	Q4	Q5
*n* (Adherence scores, range)	618	151 (3–6)	102 (7)	111 (8)	102 (9)	152 (10–12)
Mean ± SD						
Age (years)	52.37 ± 8.29	50.88 ± 8.28	52.89 ± 8.70	52.33 ± 9.13	53.01 ± 8.49	53.11 ± 7.09
BMI (kg/m^2^)	23.33 ± 2.97	24.88 ± 3.83	23.56 ± 2.61	23.41 ± 2.64	22.60 ± 2.24	22.07 ± 2.01
Physical activity (MET-hours/week)	33.02 ± 33.96	11.93 ± 11.05	21.61 ± 33.70	26.09 ± 18.97	43.00 ± 34.84	60.01 ± 37.28
Energy intake (kcal/day)	1750.11 ± 574.97	1595.37 ± 466.89	1715.65 ± 573.44	1773.18 ± 536.03	1771.97 ± 669.02	1895.45 ± 599.11
*n* (%)						
Menopausal status at diagnosis						
Premenopausal	407 (65.86)	101 (66.89)	64 (62.75)	77 (69.37)	63 (61.76)	102 (67.11)
Postmenopausal	211 (34.14)	50 (33.11)	38 (37.25)	34 (30.63)	39 (38.24)	50 (32.89)
Education level						
Elementary school or below	69 (11.20)	20 (13.33)	13 (12.75)	12 (10.90)	10 (9.80)	14 (9.21)
Middle school	65 (10.55)	17 (11.33)	10 (9.80)	13 (11.82)	12 (11.76)	13 (8.55)
High school	268 (43.51)	71 (47.33)	40 (39.21)	47 (42.73)	41 (40.20)	69 (45.40)
College or above	214 (34.74)	42 (28.01)	39 (38.24)	38 (34.55)	39 (38.24)	56 (36.84)
Marital status						
Married or cohabiting	489 (79.77)	120 (80.00)	77 (76.24)	87 (79.09)	86 (85.15)	119 (78.81)
Unmarried, divorced or widowed	124 (20.23)	30 (20.00)	24 (23.76)	23 (20.91)	15 (14.85)	32 (21.19)
Smoking status						
Never	544 (91.58)	124 (87.32)	90 (90.91)	96 (90.57)	96 (96.00)	138 (93.88)
Ever	50 (8.42)	18 (12.68)	9 (9.09)	10 (9.43)	4 (4.00)	9 (6.12)
Alcohol status						
Never	240 (39.02)	52 (34.67)	40 (39.60)	35 (31.53)	50 (49.02)	63 (41.72)
Past	235 (38.21)	61 (40.67)	34 (33.67)	43 (38.74)	36 (35.29)	61 (40.40)
Current	140 (22.77)	37 (24.66)	27 (26.73)	33 (29.73)	16 (15.69)	27 (17.88)
Dietary supplement use						
Yes	391 (63.89)	87 (58.39)	58 (56.86)	61 (55.45)	71 (70.30)	114 (76.00)
No	221 (36.11)	62 (41.61)	44 (43.14)	49 (44.55)	30 (29.70)	36 (24.00)
AJCC stage at diagnosis						
I	307 (49.68)	71 (47.02)	52 (50.98)	50 (45.05)	53 (51.96)	81 (53.29)
II	247 (39.97)	66 (43.71)	41 (40.20)	47 (42.34)	39 (38.24)	54 (35.53)
III	64 (10.35)	14 (9.27)	9 (8.82)	14 (12.61)	10 (9.80)	17 (11.18)
Time since surgery						
1–<2 years	215 (34.79)	54 (35.76)	33 (32.36)	43 (38.74)	37 (36.27)	48 (31.58)
2–<5 years	240 (38.83)	57 (37.75)	35 (34.31)	42 (37.84)	39 (38.24)	67 (44.08)
≥5 years	163 (26.38)	40 (26.49)	34 (33.33)	26 (23.42)	26 (25.49)	37 (24.34)
ER status						
Negative	151 (24.43)	36 (23.84)	25 (24.51)	27 (24.32)	28 (27.45)	35 (23.03)
Positive	467 (75.57)	115 (76.16)	77 (75.49)	84 (75.68)	74 (72.55)	117 (76.97)
PR status						
Negative	216 (34.95)	48 (31.79)	37 (36.27)	46 (41.44)	35 (34.31)	50 (32.89)
Positive	402 (65.05)	103 (68.21)	65 (63.73)	65 (58.56)	67 (65.69)	102 (67.11)

Abbreviations: SD, standard deviation; BMI, body mass index; MET, metabolic equivalent task; AJCC, American Joint Committee on Cancer; ER, estrogen receptor; PR, progesterone receptor.

**Table 3:** Least-squares means (LS-means) ^1^ and 95% confidence intervals (CIs) of HRQoL scores according to the quintiles of adherence scores among breast cancer survivors with stage I to III (*n* = 618).

	LS-Means (95% CIs) of HRQoL Scores According to Adherence Scores					*p* for Trend ^2^
	Q1	Q2	Q3	Q4	Q5	
*n* (Adherence Scores, Range)	151 (3–6)	102 (7)	111 (8)	102 (9)	152 (10–12)	
Physical component summary	46.29 (44.76–47.83)	46.70 (44.98–48.41)	48.29 (46.61–49.98)	48.41 (46.62–50.20)	48.99 (47.34–50.63)	<0.001 ^3^
Physical health sub-scales						
Physical functioning	44.08 (42.47–45.69)	45.06 (43.27–46.86)	45.83 (44.06–47.59)	47.20 (45.33–49.08)	47.30 (45.58–49.03)	<0.001 ^3^
Role-physical	45.20 (43.25–47.15)	45.86 (43.69–48.03)	47.76 (45.62–49.89)	45.90 (43.63–48.17)	46.87 (44.78–48.96)	0.13
Bodily pain	47.27 (45.25–49.29)	48.36 (46.11–50.62)	49.72 (47.50–51.93)	50.02 (47.67–52.37)	49.91 (47.74–52.08)	0.01 ^3^
General health	44.85 (42.88–46.82)	44.84 (42.64–47.04)	46.08 (43.92–48.25)	45.39 (43.10–47.68)	46.35 (44.24–48.47)	0.14
Mental component summary	44.54 (42.39–46.68)	46.15 (43.75–48.54)	46.29 (43.93–48.64)	45.32 (42.82–47.82)	45.62 (43.32–47.93)	0.51
Mental health sub-scales						
Vitality	45.37 (43.05–47.68)	47.31 (44.72–49.89)	48.19 (45.65–50.73)	47.67 (44.98–50.37)	48.56 (46.07–51.04)	0.02 ^3^
Social functioning	48.07 (46.29–49.84)	48.52 (46.54–50.50)	49.14 (47.19–51.10)	48.64 (46.57–50.71)	49.25 (47.34–51.16)	0.24
Role-emotional	43.44 (41.20–45.68)	45.49 (42.99–48.00)	44.85 (42.39–47.31)	44.54 (41.93–47.15)	44.46 (42.05–46.86)	0.60
Mental health	43.19 (40.96–45.43)	44.33 (41.85–46.82)	45.58 (43.13–48.03)	44.61 (42.01–47.21)	44.85 (42.45–47.24)	0.18

Abbreviations: LS-means, least-squares means; CIs, confidence intervals; HRQoL, health-related quality of life; ^1^ Models were adjusted for age (years; continuous), energy intake (kcal/day; continuous), menopausal status at diagnosis (premenopausal or postmenopausal status), stage (I, II, or III), time since surgery (1 to <2 years, 2 to <5 years, or ≥5 years), education level (elementary school or below, middle school, high school, or college or above), dietary supplement use (yes or no), alcohol intake (current, past, or never), smoking status (never or ever), and center.; ^2^ *p* for trend was calculated using the median value of each quintile category as a continuous variable.; ^3^ *p* for trend remained significant at α = 0.1 after adjusting for multiple comparisons with false discovery rate method.

**Table 4:** Least-squares means (LS-means) ^1^ and 95% confidence intervals (CIs) of HRQoL scores according to the quintiles of adherence scores among breast cancer survivors by stage.

	**LS-Means (95% CIs) of HRQoL Scores among Breast Cancer Survivors with Stage I (*n* = 307)**					*p* for trend ^2^
	**Q1**	**Q2**	**Q3**	**Q4**	**Q5**	
*n* (Adherence scores, range)	71 (3–6)	52 (7)	50 (8)	53 (9)	81 (10–12)	
Physical component summary ^3^	48.06(45.99–50.13)	49.09(46.81–51.38)	49.38(47.03–51.73)	49.46(47.12–51.81)	48.61(46.53–50.69)	0.60
Physical health sub-scales						
Physical functioning	45.74 (43.56–47.92)	48.53 (46.11–50.94)	47.88 (45.40–50.35)	47.91 (45.44–50.38)	48.21 (46.02–50.41)	0.08
Role-physical ^3^	48.66 (46.11–51.21)	48.59 (45.76–51.42)	49.60 (46.70–52.51)	48.67 (45.78–51.57)	47.79 (45.22–50.36)	0.56
Bodily pain ^3^	48.41 (45.46–51.37)	49.41 (46.14–52.69)	49.72 (46.36–53.08)	49.93 (46.58–53.28)	48.22 (45.24–51.19)	0.96
General health	44.28 (41.64–46.92)	44.82 (41.90–47.74)	45.21 (42.21–48.20)	44.42 (41.43–47.41)	44.91 (42.25–47.56)	0.75
Mental component summary	44.87 (42.08–47.67)	45.95 (42.85–49.05)	46.08 (42.90–49.26)	44.24 (41.07–47.41)	45.43 (42.61–48.25)	>0.99
Mental health sub-scales						
Vitality	45.12 (41.85–48.38)	46.42 (42.80–50.04)	46.88 (43.17–50.60)	45.75 (42.05–49.46)	46.65 (43.36–49.94)	0.49
Social functioning ^3^	49.96 (47.63–52.30)	50.48 (47.89–53.06)	50.72 (48.07–53.37)	48.92 (46.27–51.56)	49.67 (47.32–52.02)	0.55
Role-emotional	45.36 (42.40–48.31)	47.65 (44.37–50.93)	45.97 (42.61–49.34)	45.58 (42.23–48.94)	44.85 (41.87–47.83)	0.46
Mental health	43.12 (40.04–46.21)	43.89 (40.47–47.31)	45.33 (41.83–48.84)	43.23 (39.74–46.73)	45.08 (41.97–48.19)	0.33
	**LS-Means (95% CIs) of HRQoL Scores among Breast Cancer Survivors with Stage II–III (*n* = 311)**					*p* for trend ^2^
*n* (Adherence scores, range)	80 (3–6)	50 (7)	61 (8)	49 (9)	71 (10–12)	
Physical component summary ^3^	45.70 (43.46–47.94)	45.30 (42.80–47.80)	48.59 (46.16–51.02)	48.49 (45.82–51.15)	50.68 (48.11–53.25)	<0.001 ^4^
Physical health sub-scales						
Physical functioning	44.19 (41.88–46.51)	43.12 (40.53–45.72)	45.65 (43.14–48.17)	48.23 (45.47–50.99)	48.16 (45.50–50.82)	<0.001 ^4^
Role-physical ^3^	43.04 (40.11–45.96)	44.03 (40.75–47.31)	47.21 (44.02–50.39)	44.03 (40.54–47.52)	46.75 (43.39–50.12)	0.03 ^4^
Bodily pain ^3^	47.20 (44.48–49.91)	48.19 (45.15–51.22)	51.05 (48.09–54.00)	51.41 (48.18–54.65)	53.07 (49.95–56.19)	<0.001 ^4^
General health	45.53 (42.58–48.48)	44.66 (41.36–47.96)	47.31 (44.10–50.52)	46.50 (42.98–50.02)	47.41 (44.02–50.80)	0.17
Mental component summary	44.73 (41.43–48.03)	46.32 (42.63–50.02)	46.91 (43.32–50.49)	46.79 (42.85–50.72)	45.40 (41.61–49.19)	0.56
Mental health sub-scales						
Vitality	45.97 (42.65–49.29)	48.14 (44.42–51.86)	49.81 (46.20–53.42)	49.65 (45.69–53.61)	50.50 (46.68–54.31)	0.01 ^4^
Social functioning ^3^	46.96 (44.29–49.64)	47.04 (44.05–50.04)	48.41 (45.50–51.31)	49.05 (45.86–52.23)	49.01 (45.94–52.08)	0.09
Role-emotional	42.93 (39.51–46.35)	44.27 (40.44–48.10)	45.24 (41.53–48.96)	44.82 (40.74–48.90)	44.95 (41.02–48.87)	0.24
Mental health	43.88 (40.62–47.15)	44.78 (41.13–48.44)	46.21 (42.66–49.76)	46.53 (42.64–50.42)	44.19 (40.44–47.94)	0.51

Abbreviations: LS-means, least-squares means; CIs, confidence intervals; HRQoL, health-related quality of life; ^1^ Models were adjusted for age (years; continuous), energy intake (kcal/day; continuous), menopausal status at diagnosis (premenopausal or postmenopausal status), time since surgery (1 to <2 years, 2 to <5 years, or ≥5 years), education level (elementary school or below, middle school, high school, or college or above), dietary supplement use (yes or no), alcohol intake (current, past, or never), smoking status (never or ever), and center. In the analysis of stage II–III, we additionally adjusted for stage (II or III); ^2^ *p* for trend was calculated using the median value of each quintile category as a continuous variable; ^3^ *p* for interaction was statistically significant (<0.05); ^4^ *p* for trend remained significant at α = 0.1 after adjusting for multiple comparisons with false discovery rate method.

**Table 5:** Least-squares means (LS-means) ^1^ and 95% confidence intervals (CIs) of HRQoL scores according to the quintiles of physical activity levels among breast cancer survivors with stage I to III (*n* = 618).

	LS-Means (95% CIs) of HRQoL Scores According to Physical Activity Levels					*p* for Trend ^3^
	Q1	Q2	Q3	Q4	Q5	
*n* (Physical Activity, Median) ^2^	125 (4.00)	121 (13.60)	125 (24.50)	124 (39.72)	123 (73.00)	
Physical component summary	46.93 (45.35–48.51)	46.89 (45.24–48.55)	47.14 (45.48–48.81)	48.02 (46.32–49.71)	49.35 (47.64–51.06)	0.001 ^4^
Physical health sub-scales						
Physical functioning	44.93 (43.27–46.60)	44.88 (43.13–46.63)	45.69 (43.94–47.44)	45.70 (43.92–47.48)	47.47 (45.67–49.26)	0.003 ^4^
Role-physical	46.61 (44.61–48.62)	45.92 (43.82–48.02)	45.52 (43.41–47.63)	45.70 (43.56–47.85)	47.33 (45.16–49.50)	0.34
Bodily pain	48.09 (46.02–50.17)	48.62 (46.44–50.80)	47.67 (45.49–49.86)	50.04 (47.82–52.26)	50.37 (48.12–52.61)	0.02 ^4^
General health	45.62 (43.63–47.62)	43.69 (41.60–45.79)	44.09 (41.99–46.19)	46.35 (44.22–48.49)	48.03 (45.87–50.18)	0.001 ^4^
Mental component summary	46.23 (44.03–48.42)	44.32 (42.02–46.63)	44.04 (41.73–46.35)	45.92 (43.57–48.27)	47.02 (44.65–49.39)	0.10
Mental health sub-scales						
Vitality	46.52 (44.16–48.88)	45.39 (42.90–47.87)	46.71 (44.22–49.20)	48.33 (45.80–50.86)	50.03 (47.47–52.58)	<0.001 ^4^
Social functioning	49.25 (47.44–51.06)	47.52 (45.62–49.42)	47.28 (45.37–49.18)	49.45 (47.51–51.39)	49.92 (47.96–51.87)	0.06
Role-emotional	44.84 (42.54–47.14)	44.44 (42.03–46.86)	43.31 (40.89–45.73)	43.85 (41.39–46.32)	45.77 (43.28–48.26)	0.34
Mental health	45.17 (42.89–47.45)	42.95 (40.55–45.34)	42.98 (40.58–45.39)	44.98 (42.53–47.42)	45.82 (43.35–48.29)	0.11

Abbreviations: LS-means, least-squares means; CIs, confidence intervals; HRQoL, health-related quality of life; ^1^ Models were adjusted for age (years; continuous), energy intake (kcal/day; continuous), menopausal status at diagnosis (premenopausal or postmenopausal status), stage (I, II, or III), time since surgery (1 to <2 years, 2 to <5 years, and ≥5 years), education level (elementary school or below, middle school, high school, or college or above), dietary supplement use (yes or no), alcohol intake (current, past, or never), smoking status (never or ever), and center.; ^2^ Physical activity levels (MET-hours/week); ^3^ *p* for trend was calculated using the median value of each quintile category as a continuous variable.; ^4^ *p* for trend remained significant at α = 0.1 after adjusting for multiple comparisons with false discovery rate method.

**Table 6:** Least-squares means (LS-means) ^1^ and 95% confidence intervals (CIs) of HRQoL scores according to the quintiles of physical activity levels among breast cancer survivors by stage.

	**LS-Means (95% CIs) of HRQoL Scores among Breast Cancer Survivors with Stage I (*n* = 307)**					*p* for trend ^3^
	**Q1**	**Q2**	**Q3**	**Q4**	**Q5**	
*n* (Physical activity, median) ^2^	51 (3.68)	60 (13.70)	72 (24.45)	62 (40.63)	62 (70.10)	
Physical component summary	48.73 (46.43–51.03)	47.94 (45.79–50.10)	48.62 (46.48–50.76)	47.92 (45.69–50.15)	50.60 (48.43–52.76)	0.07
Physical health sub-scales						
Physical functioning	46.30 (43.85–48.75)	46.90 (44.61–49.19)	47.98 (45.70–50.27)	46.96 (44.58–49.33)	49.14 (46.83–51.45)	0.04
Role-physical	50.76 (47.96–53.56)	48.15 (45.53–50.77)	47.45 (44.84–50.06)	45.92 (43.20–48.64)	50.20 (47.56–52.85)	0.98
Bodily pain	49.42 (46.13–52.71)	48.48 (45.40–51.55)	47.11 (44.05–50.18)	48.46 (45.27–51.65)	51.13 (48.03–54.23)	0.14
General health	45.38 (42.47–48.28)	42.78 (40.06–45.50)	43.74 (41.04–46.45)	44.25 (41.43–47.06)	47.42 (44.69–50.16)	0.02
Mental component summary	47.28 (44.21–50.34)	44.05 (41.18–46.92)	42.64 (39.78–45.49)	44.69 (41.72–47.67)	47.78 (44.89–50.67)	0.14
Mental health sub-scales						
Vitality	45.90 (42.30–49.49)	43.61 (40.25–46.97)	44.94 (41.59–48.29)	46.41 (42.92–49.90)	49.80 (46.41–53.19)	0.003 ^4^
Social functioning	52.62 (50.08–55.15)	48.53 (46.16–50.91)	47.36 (44.99–49.72)	50.19 (47.73–52.65)	51.37 (48.98–53.76)	0.42
Role-emotional	47.51 (44.25–50.78)	46.29 (43.23–49.34)	43.66 (40.62–46.71)	43.11 (39.95–46.28)	47.54 (44.46–50.62)	0.87
Mental health	45.33 (41.92–48.75)	42.50 (39.31–45.70)	42.04 (38.86–45.22)	43.89 (40.58–47.20)	46.75 (43.53–49.97)	0.08
	**LS-Means (95% CIs) of HRQoL Scores among Breast Cancer Survivors with Stage II–III (*n* = 311)**					*p* for trend ^3^
*n* (Physical activity, median) ^2^	74 (4.00)	61 (13.60)	53 (24.50)	62 (38.50)	61 (74.47)	
Physical component summary	46.17 (43.95–48.39)	46.81 (44.24–49.38)	46.82 (44.26–49.39)	49.19 (46.61–51.78)	49.13 (46.45–51.80)	0.01 ^4^
Physical health sub-scales						
Physical functioning	44.71 (42.40–47.02)	44.64 (41.97–47.32)	45.16 (42.49–47.83)	46.11 (43.41–48.80)	47.11 (44.33–49.89)	0.04 ^4^
Role-physical	44.08 (41.20–46.96)	44.88 (41.53–48.22)	44.69 (41.35–48.02)	45.89 (42.53–49.25)	45.02 (41.54–48.49)	0.56
Bodily pain	47.86 (45.19–50.53)	49.35 (46.26–52.44)	49.45 (46.36–52.54)	52.72 (49.61–55.83)	50.80 (47.58–54.01)	0.03 ^4^
General health	45.83 (43.00–48.65)	44.69 (41.41–47.96)	44.38 (41.12–47.65)	48.72 (45.42–52.01)	48.55 (45.15–51.95)	0.01 ^4^
Mental component summary	45.70 (42.49–48.91)	45.21 (41.50–48.93)	45.88 (42.17–49.59)	46.94 (43.20–50.68)	46.05 (42.19–49.92)	0.67
Mental health sub-scales						
Vitality	47.21 (43.96–50.45)	47.52 (43.76–51.27)	48.66 (44.91–52.42)	49.93 (46.15–53.71)	50.13 (46.23–54.04)	0.08
Social functioning	47.11 (44.51–49.71)	47.37 (44.36–50.38)	48.14 (45.14–51.15)	48.79 (45.76–51.82)	48.86 (45.73–51.99)	0.19
Role-emotional	43.63 (40.30–46.95)	43.85 (40.00–47.70)	44.31 (40.47–48.16)	45.44 (41.57–49.31)	44.77 (40.77–48.77)	0.47
Mental health	45.24 (42.06–48.42)	44.17 (40.48–47.85)	44.34 (40.66–48.01)	46.10 (42.39–49.80)	44.60 (40.77–48.43)	0.99

Abbreviations: LS-means, least-squares means; CIs, confidence intervals; HRQoL, health-related quality of life; ^1^ Models were adjusted for age (years; continuous), energy intake (kcal/day; continuous), menopausal status at diagnosis (premenopausal or postmenopausal status status), time since surgery (1 to <2 years, 2 to <5 years, or ≥5 years), education level (elementary school or below, middle school, high school, or college or above), dietary supplement use (yes or no), alcohol intake (current, past, or never), smoking status (never or ever), and center. In the analysis of stage II–III, we additionally adjusted for stage (II or III); ^2^ Physical activity levels (MET-hours/week; continuous); ^3^ *p* for trend was calculated using the median value of each quintile category as a continuous variable; ^4^ *p* for trend remained significant at α = 0.1 after adjusting for multiple comparisons with false discovery rate method.

## Supplementary Materials

The following are available online at https://www.mdpi.com/2072-6643/11/12/2924/s1: Table S1: Least-squares means (LS-means) and 95% confidence intervals (CIs) of HRQoL scores according to the quintiles of adherence scores among breast cancer survivors by menopausal status at diagnosis; Table S2: Least-squares means (LS-means) and 95% confidence intervals (CIs) of HRQoL scores according to the quintiles of physical activity levels among breast cancer survivors by menopausal status at diagnosis; Table S3: Least-squares means (LS-means) and 95% confidence intervals (CIs) of HRQoL scores according to BMI scores among breast cancer survivors by stage; Table S4: Least-squares means (LS-means) and 95% confidence intervals (CIs) of HRQoL scores according to the quintiles of diet scores among breast cancer survivors by stage.
