# Supplementary material for: Adherence to the American Cancer Society Guidelines for Cancer Survivors and Health-Related Quality of Life among Breast Cancer Survivors
Source: Nutrients. 2019 Dec 3;11(12):2924. doi: 10.3390/nu11122924 (PMC6950022; doi:10.3390/nu11122924)
Supplement: Supplementary file 1 [file nutrients-11-02924-s001.pdf]

Table S1. Least-squares means (LS-means)[1] and 95% confidence intervals (CIs) of HRQoL scores according to the quintiles of adherence scores among breast cancer survivors by menopausal status at diagnosis

	**LS-means (95% CI) of HRQoL Scores among Breast Cancer Survivors with Premenopausal Status (*n*=407)**					
	**Q1**	**Q2**	**Q3**	**Q4**	**Q5**	*p* for trend[2]
n (Adherence scores, range)	101 (3–6)	64 (7)	77 (8)	63 (9)	102 (10–12)	
Physical component summary	46.29 (44.47–48.11)	47.98 (45.92–50.04)	49.23 (47.21–51.24)	48.67 (46.51–50.82)	49.57 (47.59–51.55)	0.001[3]
Physical health sub-scales						
Physical functioning	44.51 (42.61–46.41)	46.18 (44.03–48.32)	47.11 (45.01–49.21)	48.73 (46.48–50.98)	48.47 (46.41–50.53)	<0.001[3]
Role-physical	44.39 (42.04–46.73)	45.79 (43.14–48.44)	47.41 (44.82–50.00)	45.36 (42.58–48.14)	46.33 (43.79–48.88)	0.17
Bodily pain	47.22 (44.83–49.61)	49.86 (47.16–52.56)	50.02 (47.38–52.66)	49.42 (46.59–52.26)	50.28 (47.69–52.87)	0.04[3]
General health	46.34 (44.03–48.65)	47.37 (44.77–49.98)	49.29 (46.75–51.84)	46.42 (43.69–49.15)	47.71 (45.21–50.21)	0.39
Mental component summary	45.04 (42.41–47.68)	46.64 (43.65–49.62)	46.98 (44.06–49.89)	45.66 (42.54–48.78)	45.84 (42.98–48.70)	0.73
Mental health sub-scales						
Vitality	44.57 (41.78–47.37)	47.84 (44.68–50.99)	47.97 (44.88–51.06)	47.26 (43.95–50.57)	49.08 (46.05–52.11)	0.01[3]
Social functioning	48.87 (46.74–51.00)	49.15 (46.74–51.56)	50.07 (47.71–52.43)	49.10 (46.58–51.63)	48.99 (46.68–51.30)	0.91
Role-emotional	42.95 (40.23–45.66)	45.63 (42.56–48.70)	45.07 (42.06–48.07)	43.97 (40.76–47.19)	43.88 (40.93–46.82)	0.78
Mental health	44.66 (42.02–47.30)	45.50 (42.52–48.48)	47.09 (44.18–50.01)	46.26 (43.14–49.39)	46.24 (43.38–49.11)	0.22
	**LS-means (95% CI) of HRQoL Scores among Breast Cancer Survivors with Postmenopausal Status (*n*=211)**					*p* for trend[2]
n (Adherence scores, range)	50 (3–6)	38 (7)	34 (8)	39 (9)	50 (10–12)	
Physical component summary	46.45 (43.26–49.65)	44.16 (40.62–47.69)	46.03 (42.65–49.41)	47.86 (44.34–51.38)	47.80 (44.61–50.99)	0.12
Physical health sub-scales						
Physical functioning	43.39 (40.00–46.79)	42.52 (38.76–46.28)	43.01 (39.42–46.61)	44.76 (41.02–48.50)	45.73 (42.33–49.12)	0.09
Role-physical	47.33 (43.39–51.28)	46.04 (41.68–50.41)	47.89 (43.72–52.07)	47.68 (43.33–52.02)	47.85 (43.90–51.79)	0.60
Bodily pain	46.74 (42.54–50.94)	44.44 (39.79–49.09)	47.97 (43.53–52.41)	49.99 (45.37–54.62)	48.29 (44.10–52.49)	0.12
General health	42.97 (38.84–47.09)	41.32 (36.75–45.88)	40.24 (35.88–44.60)	43.55 (39.01–48.09)	44.75 (40.63–48.87)	0.26
Mental component summary	43.21 (38.90–47.52)	44.49 (39.73–49.26)	44.13 (39.57–48.68)	44.47 (39.72–49.21)	45.21 (40.91–49.51)	0.40
Mental health sub-scales						
Vitality	45.04 (40.33–49.75)	44.57 (39.35–49.78)	46.81 (41.82–51.79)	46.85 (41.66–52.04)	45.70 (41.00–50.41)	0.55
Social functioning	46.18 (42.52–49.85)	46.53 (42.47–50.58)	46.57 (42.70–50.45)	47.59 (43.55–51.62)	49.77 (46.11–53.43)	0.05
Role-emotional	44.55 (40.02–49.07)	44.81 (39.80–49.81)	43.29 (38.50–48.08)	45.82 (40.84–50.80)	46.00 (41.48–50.52)	0.47
Mental health	40.73 (35.96–45.50)	41.92 (36.64–47.20)	42.79 (37.74–47.83)	41.93 (36.67–47.18)	42.85 (38.08–47.61)	0.41

Abbreviations: LS-means, least-squares means; CIs, confidence intervals; HRQoL, health-related quality of life; [1] Models were adjusted for age (years; continuous), energy intake (kcal/day; continuous), stage (I, II, or III), time since surgery (1 to <2 years, 2 to <5 years, or ≥5 years), education level (elementary school or below, middle school, high school, or college or above), dietary supplement use (yes or no), alcohol intake (current, past, or never), smoking status (never or ever), and center.; [2] *p* for trend was calculated using the median value of each quintile category as a continuous variable.; [3] *p* for trend remained significant at α=0.1 after adjusting for multiple comparisons with FDR method.

Table S2. Least-squares means (LS-means)[1] and 95% confidence intervals (CIs) of HRQoL scores according to the quintiles of physical activity levels among breast cancer survivors by menopausal status at diagnosis

	LS-means (95% CI) of HRQoL Scores among Breast Cancer Survivors with Premenopausal Status (*n*=407)					*p* for trend[3]
	**Q1**	**Q2**	**Q3**	**Q4**	**Q5**	
n (Physical activity, median)[2]	83 (3.68)	83 (14.00)	74 (23.78)	85 (39.69)	82 (69.09)	
Physical component summary	46.94 (45.07–48.80)	47.74 (45.77–49.70)	48.13 (46.04–50.23)	48.85 (46.79–50.92)	49.59 (47.57–51.62)	0.01[4]
Physical health sub-scales						
Physical functioning	45.27 (43.31–47.22)	46.52 (44.45–48.58)	46.53 (44.34–48.73)	46.96 (44.80–49.13)	48.50 (46.38–50.62)	0.01[4]
Role-physical	45.99 (43.59–48.39)	45.61 (43.08–48.14)	44.93 (42.24–47.62)	45.66 (43.01–48.31)	45.80 (43.20–48.40)	0.98
Bodily pain	47.93 (45.49–50.37)	49.09 (46.52–51.66)	48.86 (46.12–51.59)	49.94 (47.24–52.63)	50.56 (47.91–53.20)	0.06
General health	46.77 (44.43–49.12)	46.57 (44.10–49.04)	46.57 (43.94–49.20)	48.42 (45.83–51.02)	48.94 (46.40–51.48)	0.03[4]
Mental component summary	46.37 (43.69–49.05)	45.76 (42.94–48.58)	44.20 (41.19–47.20)	46.13 (43.17–49.09)	46.59 (43.69–49.49)	0.62
Mental health sub-scales						
Vitality	45.51 (42.67–48.36)	46.19 (43.19–49.19)	46.88 (43.69–50.07)	48.46 (45.31–51.61)	49.39 (46.30–52.47)	0.01[4]
Social functioning	49.73 (47.58–51.89)	48.62 (46.35–50.89)	47.45 (45.04–49.87)	49.88 (47.50–52.26)	49.83 (47.50–52.17)	0.42
Role-emotional	44.45 (41.69–47.22)	44.85 (41.93–47.76)	42.62 (39.52–45.72)	43.68 (40.62–46.74)	44.41 (41.42–47.41)	0.91
Mental health	46.03 (43.35–48.71)	45.43 (42.60–48.26)	44.31 (41.30–47.31)	45.89 (42.92–48.86)	46.66 (43.76–49.57)	0.45
	**LS-means (95% CI) of HRQoL Scores among Breast Cancer Survivors with Postmenopausal Status (*n*=211)**					*p* for trend[3]
n (Physical activity, median)[2]	42 (4.96)	38 (12.76)	51 (24.50)	39 (40.75)	41 (74.47)	
Physical component summary	47.51 (44.31–50.70)	45.55 (42.15–48.95)	45.36 (42.15–48.57)	46.25 (42.83–49.68)	49.21 (45.75–52.67)	0.07
Physical health sub-scales						
Physical functioning	44.52 (41.12–47.93)	42.21 (38.58–45.84)	44.04 (40.62–47.46)	43.62 (39.97–47.27)	45.99 (42.30–49.67)	0.15
Role-physical	48.36 (44.46–52.26)	47.40 (43.24–51.55)	46.54 (42.62–50.45)	45.65 (41.46–49.83)	50.54 (46.31–54.76)	0.18
Bodily pain	48.25 (44.05–52.44)	47.42 (42.95–51.89)	44.41 (40.19–48.62)	49.39 (44.89–53.89)	49.54 (45.00–54.08)	0.18
General health	44.71 (40.74–48.68)	38.47 (34.24–42.71)	40.57 (36.58–44.56)	43.61 (39.35–47.87)	47.03 (42.72–51.33)	0.005[4]
Mental component summary	45.32 (41.08–49.55)	41.48 (36.97–45.99)	42.74 (38.49–46.99)	45.55 (41.01–50.09)	47.24 (42.65–51.82)	0.05
Mental health sub-scales						
Vitality	47.11 (42.49–51.74)	42.55 (37.62–47.48)	44.57 (39.93–49.22)	46.26 (41.30–51.23)	49.66 (44.65–54.67)	0.03
Social functioning	48.20 (44.54–51.86)	45.42 (41.52–49.32)	46.27 (42.60–49.95)	48.32 (44.40–52.25)	49.82 (45.85–53.78)	0.07
Role-emotional	45.16 (40.67–49.66)	44.12 (39.34–48.91)	43.61 (39.10–48.12)	44.19 (39.37–49.01)	48.41 (43.55–53.28)	0.07
Mental health	43.55 (38.85–48.24)	38.61 (33.61–43.62)	40.33 (35.62–45.05)	44.32 (39.28–49.35)	44.04 (38.96–49.13)	0.15

Abbreviations: LS-means, least-squares means; CIs, confidence intervals; HRQoL, health-related quality of life; [1] Models were adjusted for age (years; continuous), energy intake (kcal/day; continuous), stage (I, II, or III), time since surgery (1 to <2 years, 2 to <5 years, or ≥5 years), education level (elementary school or below, middle school, high school, or college or above), dietary supplement use (yes or no), alcohol intake (current, past, or never), smoking status (never or ever), and center.; [2] Physical activity levels (MET-hours/week; continuous); [3] *p* for trend was calculated using the median value of each quintile category as a continuous variable.; [4] *p* for trend remained significant at α=0.1 after adjusting for multiple comparisons with FDR method.

Table S3. Least-squares means (LS-means)[1] and 95% confidence intervals (CIs) of HRQoL scores according to BMI scores[2] among breast cancer survivors by stage

	LS-means (95% CI) of HRQoL Scores among Breast Cancer Survivors with Stage I (*n*=307)				*p* for trend[4]
	**1**	**2**	**3**	**4**	
n (BMI, median)	18 (18.06, 31.65)[3]	67 (26.45)	67 (23.94)	155 (21.33)	
Physical component summary[5]	50.17 (46.81–53.53)	47.64 (45.58–49.69)	49.96 (47.74–52.18)	48.92 (47.13–50.72)	0.24
Physical health sub-scales					
Physical functioning	47.40 (43.83–50.97)	46.13 (43.95–48.32)	48.75 (46.39–51.12)	48.00 (46.09–49.90)	0.25
Role-physical	51.54 (47.39–55.69)	47.08 (44.54–49.62)	49.15 (46.41–51.89)	48.83 (46.62–51.05)	0.08
Bodily pain	50.31 (45.48–55.14)	48.05 (45.09–51.01)	49.60 (46.40–52.79)	49.13 (46.55–51.71)	0.43
General health	42.99 (38.70–47.28)	45.72 (43.09–48.35)	44.89 (42.05–47.73)	44.29 (42.00–46.59)	0.16
Mental component summary	44.33 (39.76–48.90)	46.18 (43.38–48.98)	44.40 (41.38–47.42)	45.20 (42.76–47.64)	0.52
Mental health sub-scales					
Vitality[5]	47.48 (42.17–52.80)	47.29 (44.03–50.55)	46.34 (42.82–49.86)	44.99 (42.15–47.83)	0.29
Social functioning	49.52 (45.71–53.33)	49.66 (47.32–52.00)	49.40 (46.87–51.92)	50.39 (48.35–52.43)	0.55
Role-emotional	46.26 (41.41–51.11)	45.11 (42.14–48.08)	46.06 (42.85–49.26)	46.04 (43.45–48.64)	0.55
Mental health	42.15 (37.11–47.19)	44.90 (41.81–47.99)	43.06 (39.72–46.40)	44.26 (41.56–46.95)	0.59
	**LS-means (95% CI) of HRQoL Scores among Breast Cancer Survivors with Stage II–III (*n*=311)**				*p* for trend[4]
n (BMI, median)	15 (18.29, 31.04)[3]	75 (26.45)	80 (23.83)	141 (21.33)	
Physical component summary[5]	45.86 (41.85–49.88)	45.25 (42.94–47.56)	48.04 (45.70–50.37)	48.37 (46.22–50.53)	0.01[6]
Physical health sub-scales					
Physical functioning	46.22 (42.08–50.37)	43.12 (40.73–45.51)	45.65 (43.24–48.06)	46.43 (44.21–48.66)	0.04[6]
Role-physical	42.01 (36.82–47.20)	43.40 (40.41–46.39)	45.31 (42.30–48.33)	45.94 (43.15–48.72)	0.03[6]
Bodily pain	44.72 (39.85–49.59)	48.82 (46.01–51.62)	50.07 (47.24–52.90)	50.62 (48.00–53.23)	0.02[6]
General health	43.54 (38.34–48.74)	45.86 (42.87–48.86)	47.11 (44.09–50.14)	46.04 (43.25–48.83)	0.15
Mental component summary	42.16 (36.37–47.94)	47.29 (43.95–50.63)	45.64 (42.27–49.00)	45.79 (42.68–48.90)	0.93
Mental health sub-scales					
Vitality[5]	45.07 (39.17–50.97)	48.10 (44.71–51.50)	48.63 (45.20–52.06)	48.85 (45.68–52.01)	0.28
Social functioning	46.37 (41.65–51.08)	48.04 (45.32–50.76)	47.64 (44.89–50.38)	48.03 (45.50–50.56)	0.67
Role-emotional	42.32 (36.30–48.34)	44.91 (41.44–48.38)	43.83 (40.33–47.33)	44.30 (41.07–47.53)	0.88
Mental health	40.03 (34.30–45.77)	45.68 (42.37–48.98)	45.18 (41.84–48.51)	45.35 (42.27–48.42)	0.34

Abbreviations: LS-means, least-squares means; CIs, confidence intervals; HRQoL, health-related quality of life; BMI, body mass index; [1] Models were adjusted for age (years; continuous), energy intake (kcal/day; continuous), menopausal status at diagnosis (premenopausal or postmenopausal status), time since surgery (1 to <2 years, 2 to <5 years, or ≥5 years), education level (elementary school or below, middle school, high school, or college or above), dietary supplement use (yes or no), alcohol intake (current, past, or never), smoking status (never or ever), and center. In the analysis of stage II–III, we additionally adjusted for stage (II or III).; [2] BMI scores, 1 (<18.5 or ≥30 kg/m^2); 2 (25–<30 kg/m^2); 3 (23–<25 kg/m^2); 4 (18.5–<23 kg/m^2); [3] Median values for <18.5 kg/m^2 and ≥30 kg/m^2; [4] *p* for trend was calculated using the median value of each category as a continuous variable.; [5] *p* for interaction was statistically significant (<0.05).; [6] *p* for trend remained significant at α=0.1 after adjusting for multiple comparisons with false discovery rate method.

Table S4. Least-squares means (LS-means)[1] and 95% confidence intervals (CIs) of HRQoL scores according to the quintiles of diet scores[2] among breast cancer survivors by stage

	**LS-means (95% CI) of HRQoL Scores among Breast Cancer Survivors with Stage I (*n*=307)**					*p* for trend[3]
	**Q1**	**Q2**	**Q3**	**Q4**	**Q5**	
n (Diet scores, range)	49 (3–5)	45 (6)	108 (7–8)	51 (9)	54 (10–12)	
Physical component summary[4]	48.68 (46.19–51.18)	50.22 (47.92–52.52)	48.00 (46.03–49.97)	48.18 (45.86–50.51)	48.91 (46.56–51.26)	0.58
Physical health sub-scales						
Physical functioning	47.06 (44.41–49.70)	48.10 (45.66–50.53)	46.90 (44.81–48.98)	46.30 (43.84–48.76)	49.39 (46.90–51.87)	0.53
Role-physical[4]	48.20 (45.11–51.29)	50.25 (47.40–53.10)	47.88 (45.44–50.31)	48.42 (45.54–51.29)	47.90 (44.99–50.81)	0.57
Bodily pain[4]	48.54 (44.99–52.09)	51.60 (48.33–54.87)	47.73 (44.94–50.53)	49.81 (46.50–53.11)	46.79 (43.46–50.13)	0.31
General health	44.61 (41.42–47.80)	44.88 (41.93–47.82)	45.01 (42.50–47.53)	43.57 (40.60–46.54)	45.07 (42.07–48.07)	0.93
Mental component summary	44.59 (41.20–47.99)	45.90 (42.76–49.03)	44.97 (42.29–47.64)	45.91 (42.75–49.07)	44.88 (41.69–48.08)	0.84
Mental health sub-scales						
Vitality	45.73 (41.78–49.68)	46.94 (43.29–50.58)	44.70 (41.58–47.81)	46.89 (43.21–50.56)	46.94 (43.23–50.66)	0.73
Social functioning[4]	50.07 (47.24–52.89)	51.11 (48.51–53.72)	49.18 (46.95–51.40)	50.31 (47.68–52.93)	49.21 (46.55–51.87)	0.49
Role-emotional[4]	45.08 (41.49–48.68)	46.81 (43.49–50.13)	45.66 (42.82–48.49)	45.33 (41.98–48.68)	45.47 (42.08–48.85)	0.94
Mental health	42.98 (39.23–46.73)	44.59 (41.13–48.05)	43.92 (40.96–46.87)	44.69 (41.20–48.18)	43.81 (40.28–47.34)	0.65
	**LS-means (95% CI) of HRQoL Scores among Breast Cancer Survivors with Stage II–III (*n*=311)**					*p* for trend[3]
n (Diet scores, range)	59 (3–5)	45 (6)	98 (7–8)	47 (9)	62 (10–12)	
Physical component summary[4]	45.97 (43.37–48.57)	45.81 (43.17–48.45)	46.93 (44.56–49.30)	48.81 (46.09–51.52)	48.94 (46.36–51.52)	0.01[5]
Physical health sub-scales						
Physical functioning	44.60 (41.92–47.28)	43.19 (40.47–45.92)	45.17 (42.73–47.61)	46.74 (43.94–49.54)	47.30 (44.64–49.95)	0.01[5]
Role-physical[4]	42.62 (39.29–45.95)	43.07 (39.68–46.45)	45.11 (42.07–48.14)	46.97 (43.48–50.45)	46.60 (43.30–49.90)	0.01[5]
Bodily pain[4]	47.96 (44.83–51.10)	47.47 (44.29–50.65)	49.92 (47.07–52.77)	50.49 (47.22–53.76)	52.14 (49.04–55.25)	0.01[5]
General health	45.30 (41.96–48.65)	45.57 (42.17–48.96)	45.66 (42.61–48.70)	48.59 (45.10–52.08)	45.71 (42.40–49.02)	0.52
Mental component summary	44.45 (40.72–48.18)	44.57 (40.78–48.36)	45.98 (42.58–49.38)	47.91 (44.01–51.81)	47.21 (43.51–50.91)	0.10
Mental health sub-scales						
Vitality	45.50 (41.75–49.26)	47.73 (43.92–51.54)	47.09 (43.67–50.51)	51.37 (47.44–55.29)	50.30 (46.58–54.02)	0.01[5]
Social functioning[4]	47.30 (44.28–50.31)	47.18 (44.12–50.25)	46.42 (43.67–49.17)	48.59 (45.43–51.74)	49.76 (46.77–52.75)	0.05[5]
Role-emotional[4]	42.79 (38.93–46.64)	41.95 (38.03–45.87)	44.93 (41.42–48.44)	46.11 (42.09–50.14)	46.11 (42.29–49.93)	0.06[5]
Mental health	43.70 (40.01–47.40)	43.05 (39.29–46.80)	45.96 (42.60–49.32)	47.04 (43.18–50.90)	45.87 (42.21–49.53)	0.19

Abbreviations: LS-means, least-squares means; CIs, confidence intervals; HRQoL, health-related quality of life; [1] Models were adjusted for age (years; continuous), energy intake (kcal/day; continuous), menopausal status at diagnosis (premenopausal or postmenopausal status), time since surgery (1 to <2 years, 2 to <5 years, or 5 years or more), education level (elementary school or below, middle school, high school, or college or above), dietary supplement use (yes or no), alcohol intake (current, past, or never), smoking status (never or ever), and center. In the analysis of stage II–III, we additionally adjusted for stage (II or III).; [2] Diet scores were calculated by summing each score from the three food groups.; [3] *p* for trend was calculated using the median value of each quintile category as a continuous variable.; [4] *p* for interaction was statistically significant (<0.05).; [5] *p* for trend remained significant at α=0.1 after adjusting for multiple comparisons with false discovery rate method.
